# HERMES: an open-source mining tool for open-access literature

**DOI:** 10.1093/bioadv/vbag058

**Published:** 2026-02-17

**Authors:** Julien Charest, Katarina Priselac, Georg H Reischer, Andreas H Farnleitner, Robert L Mach, Astrid R Mach-Aigner

**Affiliations:** Institute of Chemical, Environmental and Bioscience Engineering, TU Wien, Wien 1060, Austria; Institute of Chemical, Environmental and Bioscience Engineering, TU Wien, Wien 1060, Austria; Institute of Chemical, Environmental and Bioscience Engineering, TU Wien, Wien 1060, Austria; Institute of Chemical, Environmental and Bioscience Engineering, TU Wien, Wien 1060, Austria; Division Water Quality and Health, Karl Landsteiner University for Health Sciences, Dr.-Karl-Dorrek, Krems an der Donau 3500, Austria; Institute of Chemical, Environmental and Bioscience Engineering, TU Wien, Wien 1060, Austria; Institute of Chemical, Environmental and Bioscience Engineering, TU Wien, Wien 1060, Austria

## Abstract

**Motivation:**

The exponential growth of open-access scientific literature presents researchers with unprecedented opportunities but also poses a significant challenge: how to efficiently identify and prioritize relevant publications in a transparent and customizable manner. Existing search engines index large volumes of biomedical literature but rarely provide user-defined ranking options, reproducibility, or integration of domain-specific criteria. This gap is particularly limiting for specialized fields, where nuanced keyword combinations, literature recency, and contextual interpretation are critical.

**Results:**

We present HERMES, an open-source literature mining tool for targeted retrieval and ranking of full-text open-access publications from PubMed Central (PMC). HERMES employs a composite scoring algorithm that integrates keyword frequency, citation counts, and publication age to prioritize publications. It further supports summarization, biomedical entity recognition, and PDF report generation. An intuitive graphical user interface (GUI) allows researchers without programming expertise to perform complex literature mining tasks, while multithreaded processing ensures efficiency for large-scale queries. HERMES provides a reproducible and adaptable framework for literature discovery, empowering researchers to rapidly identify relevant literature and promoting transparency and community-driven extension.

**Availability and implementation:**

HERMES (version 1.2) is implemented in Python (3.11). The source code is freely available on GitHub at https://github.com/julien-charest/hermes and is distributed under the GPL-3 license.

## 1 Introduction

The exponential growth of scientific publishing has fundamentally reshaped how researchers discover and synthesize knowledge. PubMed Central (PMC), the largest open-access repository of biomedical research, currently contains millions of full-text articles and continues to expand rapidly. While this wealth of information provides unprecedented opportunities, it also poses substantial challenges: researchers must navigate increasingly vast and heterogeneous bodies of literature, often under time constraints and with highly specific information needs. Identifying, prioritizing, and extracting insights from the most relevant articles is therefore a critical but non-trivial task. Traditional bibliographic databases such as PubMed, Europe PMC, and Google Scholar provide robust indexing and keyword-based retrieval functionalities. However, their ranking algorithms are generally optimized for broad accessibility rather than specialized research priorities. Results are often ordered according to citation counts, journal prestige, or generic relevance metrics, which can obscure recently published but highly pertinent articles. Moreover, these platforms offer limited support for transparent and reproducible ranking strategies, making it difficult for researchers to systematically justify or replicate their literature discovery workflows.

In response to these limitations, a variety of specialized tools have been developed to enhance biomedical literature, mining and exploration. For example, Textpresso was one of the earliest domain-specific search engines for full-text articles, introducing ontology-based keyword searches tailored to model organism literature (Müller *et al*. 2004). Although pioneering in the field of literature mining, its scope remains largely limited to curated corpora covering specific model organisms and biomedical domains, and its strong reliance on predefined ontologies and dictionaries constrains the flexibility of its application. PubTator and iTextMine extend this paradigm by enabling large-scale automated annotation of biomedical entities, including genes, proteins, diseases, chemicals, mutations, and pathways (Ren *et al*. 2018, Wei *et al*. 2024). These platforms support entity-level queries across millions of abstracts and full-text articles, demonstrating how natural language processing (NLP) can transform unstructured biomedical text into structured, machine-readable knowledge. Their outputs have proven invaluable for biocuration and for downstream computational analyses that rely on entity or relation extraction. However, both tools remain primarily focused on annotation and tagging, rather than on providing customizable, end-to-end workflows for literature discovery. In particular, they lack functionalities such as flexible ranking, contextual prioritization, or integrated summarization that would enable researchers to systematically identify and prioritize the most relevant publications for their specific needs.

In recent years, advances in machine learning and large language models (LLMs) have led to the emergence of a new generation of AI-assisted discovery platforms. Tools such as Elicit (https://elicit.com/) and Perplexity (https://www.perplexity.ai/) offer novel approaches to exploring scientific literature through conversational interfaces and evidence-linked output: Elicit demonstrates how LLMs can semi-automate systematic reviews, extracting key variables and organizing evidence into structured tables (Elicit 2025), while Perplexity combines LLMs with web search to deliver conversational, cited answers, enabling rapid exploration of academic and general knowledge (Perplexity 2023). These systems go beyond simple keyword matching, providing context-aware recommendations, rapid content summarization, and in some cases, semi-automated support for systematic reviews, thus greatly reducing the time and effort required. Despite these advantages, important limitations remain: most of these platforms are proprietary and subscription-based, which restricts access to full functionality and creates barriers to transparency and reproducibility. Their underlying algorithms cannot be fully audited, and researchers are unable to customize ranking criteria or integrate domain-specific keywords into the discovery process. Furthermore, many of these systems rely on pre-indexed corpora and focus primarily on abstract-level analysis, rather than parsing the full text of articles. As a result, while AI-assisted discovery tools demonstrate the potential of LLMs to accelerate literature exploration, their current design often limits adaptability and reproducibility for domain-specific applications in biomedical research. Beyond domain-specific platforms, several general-purpose NLP frameworks and biomedical deep learning models have been developed to support large-scale literature mining. spaCy is a widely adopted, production-ready NLP library that provides efficient tokenization, part-of-speech tagging, dependency parsing, and named entity recognition (Honnibal *et al*. 2020). Its modular architecture and extensibility have made it the foundation for multiple domain-specific extensions, including biomedical toolkits. These frameworks demonstrate the technical feasibility and power of advanced NLP for extracting structured knowledge from unstructured texts. However, their deployment typically requires substantial programming expertise, integration across multiple toolchains, and access to computational resources, which limits their accessibility for many biomedical researchers. As a result, a gap remains between the capabilities of advanced computational methods and the need for accessible, reproducible, and customizable literature mining workflows.

To address these limitations, we developed HERMES, an open-source literature mining tool that integrates targeted retrieval, customizable ranking, automated summarization, and entity extraction within a single, user-friendly platform. HERMES retrieves open-access full-text publications from PMC and supports two alternative scoring options for ranking results. The tool compiles outputs into structured, automatically generated portable document format (PDF) reports that include ranked publication tables, concise article summaries, and extracted biomedical entities. By providing these functionalities within an intuitive GUI, HERMES lowers the technical barrier to advanced literature mining, while its open-source design ensures transparency, reproducibility, and extensibility.

## 2 Methods

**Figure 1:**
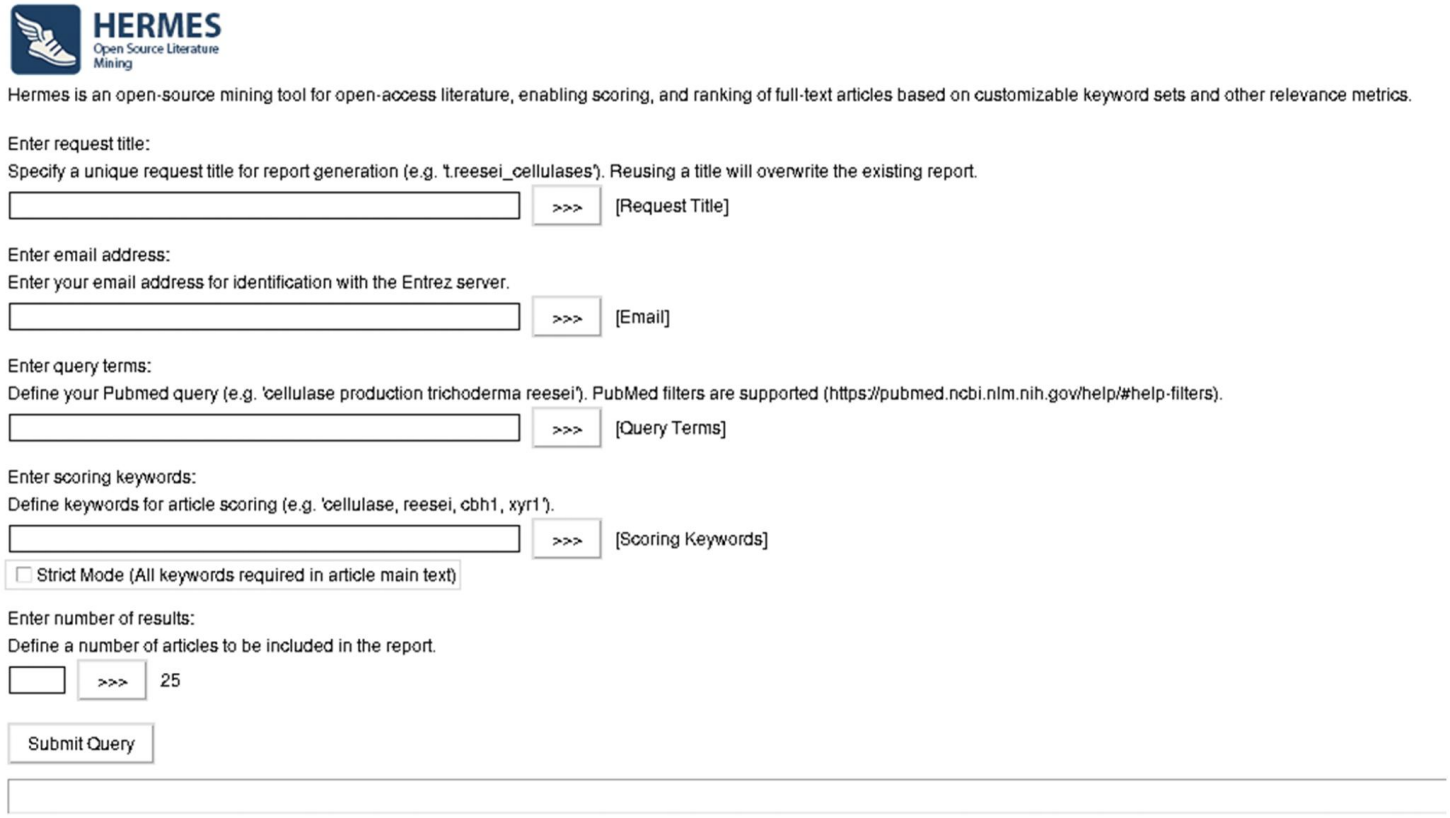
HERMES graphical user interface. The GUI provides an intuitive interface for defining and executing PMC queries. Required inputs include a request title, an email address, query terms (which may include PMC filters), scoring keywords (provided as comma-separated values), an optional “strict mode” checkbox, and the number of top-ranked results to be included in the final report. Upon submission, the user is prompted to select an output directory. During execution, a progress bar and status messages provide feedback on the query progress.

HERMES (v1.2) is a cross-platform, open-source Python application (Python 3.11) for mining open-access literature, publicly available at https://github.com/julien-charest/hermes. The application is launched via the *hermes.py* script, with core functionality organized into dedicated modules for mining, scoring, summarization, visualization, and report generation. A GUI implemented with Tkinter provides interactive configuration of queries and export parameters (Fig. 1). During processing, the interface provides real-time feedback through a progress bar and status messages. Once the analysis completes successfully and the results are saved, the GUI closes automatically, signaling completion of the workflow. For a more detailed description, please refer to the Supplementary Methods.

### 2.1 User input and corpus retrieval

The workflow starts with user-provided parameters: a request title, an NCBI-compliant email address for Entrez identification, a PMC query string, and a set of independently defined scoring keywords. Optionally, users enable a strict screening mode and select the number of top-ranked articles to include in the final report. HERMES queries the PMC database through the NCBI Entrez API to retrieve matching PMC identifiers (PMCIDs), up to a user-defined retrieval limit (retmax). For each PMCID, HERMES downloads the full XML record and parses bibliographic metadata and full-text content in a rate-limited manner compatible with NCBI usage policies. Retrieved records are consolidated into a structured results table for downstream screening and analysis.

### 2.2 Parallelized mining and robustness control

Retrieval and parsing are parallelized across PMCIDs using concurrent.futures.ThreadPoolExecutor to support corpus-scale screening on standard CPU hardware. Per-article failures are logged and automatically retried with bounded attempts to mitigate transient network or record-specific parsing issues. A run-specific log file records query parameters, processing status, and performance information.

### 2.3 Mining and relevance ranking

HERMES separates retrieval (the PMC query) from ranking (user-defined scoring keywords). Articles are screened using exact substring matching of comma-separated keywords against the parsed full text. Relevance ranking is computed using a composite scoring function that integrates (i) TF-IDF-weighted keyword evidence across the retrieved corpus, (ii) a citation-based boost, and (iii) an age-based penalty to account for recency. Each component is controlled by user-defined weights, allowing ranking behavior to be tuned to different search objectives (e.g. setting the age weight to 0 disables any recency penalty). A strict scoring mode option is available for high-specificity searches. In strict mode, the same scoring function is used, but an explicit conditional filter discards articles from ranking if any user-specified mandatory keyword is absent. All mined and scored results are consolidated and exported as a CSV file (hermes_results.csv) to enable iterative refinement and downstream analysis across multiple HERMES runs.


$$\mathrm{Score}=\frac{w_{\mathrm{Keywords}}*\sum \left(\mathrm{Keywords}\;{TF}_{i}*{\mathrm{IDF}}_{i}\right)*(w_{Ci\mathrm{tations}}*\mathrm{Citations}+1)}{w_{\mathrm{Publication}\;\mathrm{Age}}*\mathrm{Publication}\;\mathrm{Age}+1}$$

### 2.4 Summarization and named-entity extraction

For the top-ranking articles selected by the user, HERMES generates automated summaries of the parsed full text using a local LLM-based summarization routine (implemented in modules/summarizing.py; default model: google/flan-t5-large via Hugging Face). Summarization is executed concurrently across selected articles with bounded retries and can be accelerated using a GPU when available (with CPU execution supported by default). The summarization model can be substituted with alternative Hugging Face-compatible models depending on user requirements and available computational resources. Biomedical named-entity recognition (NER) is performed with spaCy and SciSpaCy (model: en_ner_bionlp13cg_md) to extract candidates for genes, proteins, diseases, chemicals, cells, organisms, tissues, and pathways. To reduce false positives, candidate entities are validated using external resolvers and ontology services.

### 2.5 Visualization and report generation

HERMES generates summary figures from the ranked dataset using matplotlib, including publication-year distributions and keyword-/score-related summaries. Outputs are organized into a structured results directory containing the run log, generated figures, the full CSV results table, and a structured PDF report. The PDF report compiles query metadata, summary figures, ranked article tables (with hyperlinks), and detailed per-article sections for the selected top results (summaries and extracted entities).

## 3 Results

### 3.1 Demonstration of HERMES usage

**Figure 2:**
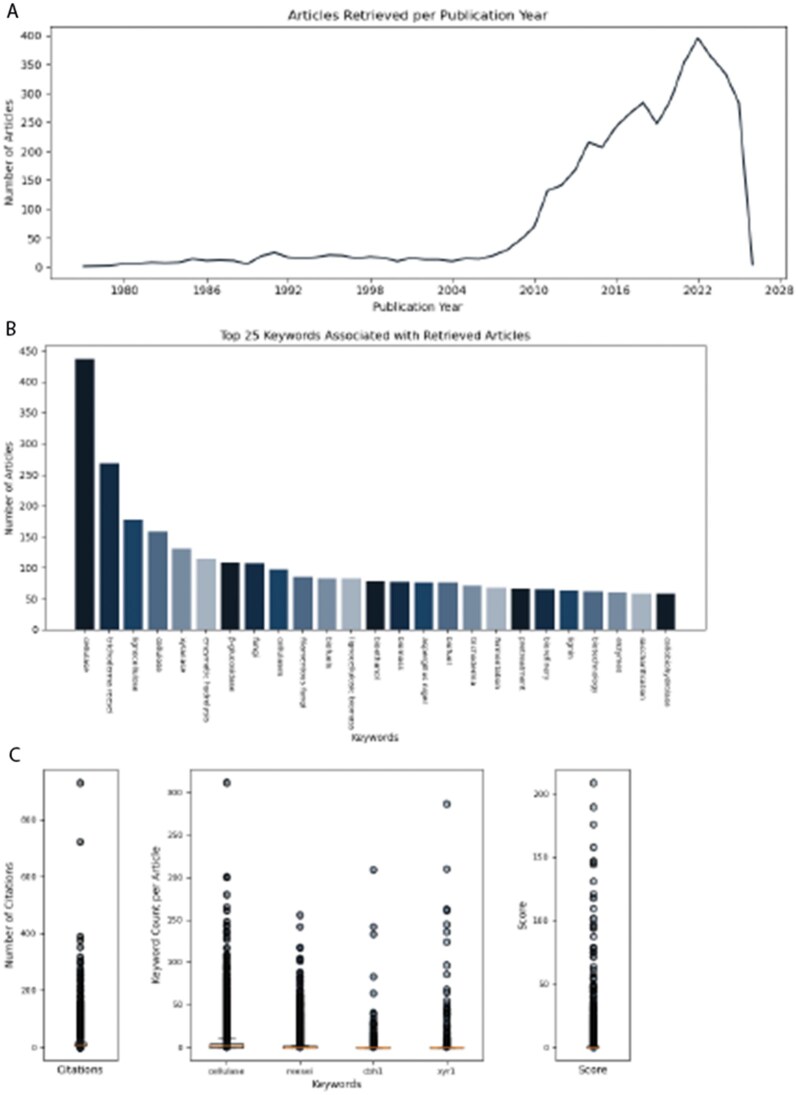
HERMES summary figures. Upon report generation, HERMES automatically produces summary visualizations: (A) the number of retrieved articles published per year, providing a temporal overview of research activity; (B) the frequency distribution of the top 25 keywords associated with the corpus, highlighting thematic trends; and (C) scoring parameter distributions, including citation counts and keyword frequencies, together with the composite HERMES score.

To illustrate HERMES in practice, we queried PMC with the search string “*cellulase production trichoderma reesei*,” retrieving 4459 open-access articles, and applied a scoring scheme based on the keywords *cellulase, reesei, cbh1*, and *xyr1* (Fig. 2). *Trichoderma reesei* is widely used in the biotechnology industry as the primary workhorse for large-scale cellulase production, making it a well-established model for enzyme regulation studies (Bhardwaj *et al*. 2021). Notably, *cbh1* encodes cellobiohydrolase I, its major secreted cellulase, while *xyr1* is the principal transcriptional activator governing cellulase gene expression (Stricker *et al*. 2008). Using both the default and strict-mode settings, HERMES automatically ranked the corpus by keyword frequency, recency, and citation counts, and generated concise summaries of each full text. Strict mode was particularly effective at prioritizing articles that highlighted the interaction between *cbh1* and *xyr1*, notably the regulation of *cbh1* expression by *xyr1*. The resulting ranked tables, summaries, and entity reports provided a compact overview of the most relevant recent studies, highlighting both core regulatory pathways and strain-specific findings. As a second test case, we queried “*ASE asymmetry C. elegans*” with scoring keywords *ASE, che-1*, and *lsy-6*, retrieving 371 open-access articles. The ASE neuron pair in *Caenorhabditis elegans* provides a classical model for neuronal asymmetry, with one neuron (ASEL) adopting a distinct fate from its contralateral counterpart (ASER) (Ortiz *et al*. 2009). HERMES identified and summarized core studies describing the role of *che-1*, a zinc-finger transcription factor that specifies ASE neuron identity, and *lsy-6*, a microRNA critical for establishing this molecular and functional left–right asymmetry (Charest *et al*. 2020). In this case, strict mode retrieved 11 articles showcasing the interaction between *che-1* and *lsy-6*, notably the regulation of *lsy-6* expression by *che-1* through transcriptional priming. The complete reports and logs for these example cases are available in the project’s GitHub repository.

### 3.2 Comparison of HERMES to LLM-based tools

Recent AI-assisted tools such as Elicit and Perplexity provide powerful, interactive ways to explore scientific literature through conversational interfaces and rapid synthesis. These tools excel at question-driven exploration, surfacing relevant papers, summaries, or cited answers with minimal user effort. However, HERMES is designed to address a complementary use case: HERMES operates directly on full-text articles retrieved from PMC, enabling screening, ranking, summarization, and entity extraction at the corpus level.

By working with complete article bodies instead of abstracts or citation snippets, HERMES allows users to apply custom keyword-driven constraints and strict inclusion rules across thousands of articles in a reproducible manner. In particular, strict mode supports high-specificity screening by discarding any article from the retrieved corpus that does not contain all user-defined keywords, which is useful for enforcing co-occurrence constraints. While keyword screening relies on exact substring matching, this can be advantageous: a term such as “toxic” will match occurrences like “toxicity” or “toxicology” when the literal substring is present, providing a simple and transparent mechanism to capture common morphological extensions.

In contrast to commercial LLM-based platforms, HERMES is fully open-source and free to use. Importantly, HERMES persists the results of the entire screening and scoring process to disk, rather than returning only a transient ranked list or conversational output. This design enables downstream data science analyses, such as re-ranking under alternative weighting schemes, integration with custom pipelines, or longitudinal comparison across multiple query iterations. In addition, HERMES generates a self-contained PDF report that includes direct hyperlinks to the retrieved articles, concise LLM-generated summaries, extracted entities, and summary figures. Together, these elements allow users to rapidly assess whether a given literature search has been fruitful and to navigate efficiently between high-level overviews and the underlying primary sources. The trade-off of this design is that HERMES prioritizes transparent, controllable, and reproducible workflows over the immediacy and flexibility of conversational interfaces. Tools like Elicit or Perplexity may be preferable for rapid hypothesis generation or exploratory questioning, whereas HERMES is better suited for systematic screening, corpus-level analysis, and reportable literature mining tasks where users require explicit control over ranking criteria and access to all intermediate results. In this sense, HERMES complements existing LLM-based tools by filling a niche at the intersection of scalable full-text literature mining, open-access data, and reproducible analysis.

Additional benchmarking analyses, framed as use-case-driven evaluations (including comparisons with selected LLM-based tools using identical queries, evaluation of NER false positives before and after validation, expert assessment of result relevance, and quantitative metrics such as precision and recall), as well as computational performance evaluations (processing time, memory usage, scalability, and comparisons of multithreaded versus single-threaded execution), are provided in the Supplementary Methods.

### 3.3 Limitations

While HERMES provides a fast and user-friendly framework for mining, prioritizing, and summarizing biomedical literature, several limitations should be noted. First, keyword-based scoring relies on exact substring matching, which may miss semantically related terms such as synonyms, acronyms, or morphological variants (e.g. “*Caenorhabditis elegans*” versus “*C. elegans*,” or “IL1B” versus “IL-1β”). Although the optional “strict mode” enforces higher specificity, both modes remain limited by the lexical surface form of the text. Second, the summarization module uses a local LLM-based approach, which generally improves coherence compared to extractive methods but may still omit fine-grained details or context; summaries should be treated as assistive overviews and key statements verified against the source text. The summarization model is configurable and can be accelerated with a GPU when available, but resource requirements may still limit throughput on some systems. Third, NER depends on the SciSpaCy BioNLP13CG model, which may underperform on entities outside its training domain or in highly specialized subfields. Nonetheless, the modular design of HERMES allows the NER model to be easily switched when improved or domain-specific alternatives become available. Additionally, NER validation against external resources reduces false positives but does not fully eliminate noise. Fourth, HERMES is currently limited to the PMC corpus (representing ∼25%–30% of PubMed-indexed records), which excludes non-open-access or subscription-based literature and may restrict coverage of certain domains. For some PMC-linked records, publisher restrictions limit full-text XML download, in which case HERMES operates on the abstract only. However, coverage is expected to increase over time as open-access publishing expands. Finally, the tool currently depends on the availability and stability of external APIs, making it sensitive to rate-limiting, downtime, or changes in API structure.

## 4 Conclusion

HERMES provides an accessible, open-source framework for mining, scoring, and summarizing biomedical literature from PMC. By integrating keyword-based scoring, automated summarization, and NER with validation against external biomedical knowledge bases, the platform enables users to efficiently extract and organize relevant information from large corpora. The graphical user interface further lowers the barrier for non-specialists, making HERMES a practical tool for streamlined literature exploration and reporting.

## Supplementary Material

vbag058_Supplementary_Data

## Data Availability

The source code of HERMES (version 1.1) is available at https://github.com/julien-charest/hermes. Full-text articles analyzed in this study were retrieved from PMC (https://www.ncbi.nlm.nih.gov/pmc/). Extracted biological entities (genes, proteins, diseases, chemicals, cells, organisms, tissues and pathways) were validated against the public databases: MyGene.info (http://mygene.info/), MyDisease.info (http://mydisease.info/), MyChem.info (https://mychem.info/), EBI OLS4 (https://www.ebi.ac.uk/ols4), and MyPathway.info (https://mypathway.info/).

## Availability and requirement

Project name: HERMES

Project home page: https://github.com/julien-charest/hermes

Operating system: Platform independent

Programming language: Python (≥3.9)

Dependencies: BioPython, BeautifulSoup4, lxml, pandas, numpy, requests, NLTK, Sumy, spaCy, SciSpacy, FPDF, matplotlib

Other requirements: Internet connection for PMC, MyGene.info, MyDisease.info, MyChem.info, EBI OLS4 and MyPathway, info queries.

License: GPL-3

Any restriction to use by non-academics: None
