# Supplementary material for: HERMES: an open-source mining tool for open-access literature
Source: Bioinform Adv. 2026 Feb 17;6(1):vbag058. doi: 10.1093/bioadv/vbag058 (PMC12952204; doi:10.1093/bioadv/vbag058)
Supplement: vbag058_Supplementary_Data [file vbag058_supplementary_data.docx]

**HERMES: an open-source mining tool for open-access literature**

**Supplemental Methods**

Julien Charest^1^, Katarina Priselac^1^, Georg H. Reischer^1^, Andreas H. Farnleitner^1,2^, Robert L. Mach^1^, Astrid R. Mach-Aigner^1^

^1^ Institute of Chemical, Environmental and Bioscience Engineering, TU Wien, Gumpendorfer Strasse 1a, 1060 Wien, Austria

^2^ Division Water Quality and Health, Karl Landsteiner University for Health Sciences, Dr.-Karl-Dorrek Straße 30, 3500 Krems an der Donau, Austria

Email addresses:

JC: julien.charest@tuwien.ac.at
KP: katarina.priselac@tuwien.ac.at
GHR: georg.reischer@tuwien.ac.at
AHF: andreas.farnleitner@tuwien.ac.at
RLM: robert.mach@tuwien.ac.at
ARMA: astrid.mach-aigner@tuwien.ac.at

**Corresponding author**

Correspondence to Julien Charest (julien.charest@tuwien.ac.at).

Table of Contents

[Installation 5](#_Toc218862877)

[Main script overview (hermes.py) and global configuration 6](#_Toc218862878)

[**NCBI Entrez access and retrieval scope** 6](#_Toc218862879)

[**Performance and parallelization** 6](#_Toc218862880)

[**Summarization configuration** 6](#_Toc218862881)

[User-defined parameters (GUI inputs) 7](#_Toc218862882)

[**Request title** 7](#_Toc218862883)

[**Email address (Entrez identification)** 7](#_Toc218862884)

[**Query terms (PMC query)** 7](#_Toc218862885)

[**Scoring keywords** 7](#_Toc218862886)

[**Strict mode** 7](#_Toc218862887)

[**Number of results (report size)** 7](#_Toc218862888)

[Mining module (modules/mining.py) 8](#_Toc218862889)

[**Record retrieval (fetch_record)** 8](#_Toc218862890)

[**XML parsing and metadata extraction (mine_pmcid)** 8](#_Toc218862891)

[**Citation count retrieval (get_citations)** 8](#_Toc218862892)

[**Full-text extraction and keyword statistics** 8](#_Toc218862893)

[**Returned data structure** 8](#_Toc218862894)

[**Customization** 8](#_Toc218862895)

[Scoring module (modules/scoring.py) 9](#_Toc218862896)

[**Term frequency and inverse document frequency** 9](#_Toc218862897)

[**Composite relevance score** 9](#_Toc218862898)

[**Effect of weighting parameters** 9](#_Toc218862899)

[**Strict mode filtering** 10](#_Toc218862900)

[Summarizing module (modules/summarizing.py) 11](#_Toc218862901)

[**Model configuration and compute device** 11](#_Toc218862902)

[**XML retrieval and preprocessing** 11](#_Toc218862903)

[**Section extraction and summary generation** 11](#_Toc218862904)

[**Figure caption extraction** 11](#_Toc218862905)

[**Named-entity recognition and validation** 11](#_Toc218862906)

[**Returned output structure** 12](#_Toc218862907)

[**Customization points** 12](#_Toc218862908)

[Visualization module (modules/generating_graphs.py) 13](#_Toc218862909)

[**Publication-year distribution** 13](#_Toc218862910)

[**Associated keyword frequency** 13](#_Toc218862911)

[**Summary statistics overview** 13](#_Toc218862912)

[**Customization** 13](#_Toc218862913)

[Report generation module (modules/generating_report.py) 14](#_Toc218862914)

[**Report structure and layout** 14](#_Toc218862915)

[**Robustness and output writing** 14](#_Toc218862916)

[**Customization** 14](#_Toc218862917)

[Utility and helper functions module (modules/adding_functions.py) 15](#_Toc218862918)

[**Resource path resolution** 15](#_Toc218862919)

[**Output directory management** 15](#_Toc218862920)

[**String sanitization for safe output** 15](#_Toc218862921)

[**Role within the HERMES architecture** 15](#_Toc218862922)

[Output directory structure and generated results 16](#_Toc218862923)

[**Top-level report directory** 16](#_Toc218862924)

[**results/ directory** 16](#_Toc218862925)

[**figures/ directory** 16](#_Toc218862926)

[**Design rationale** 16](#_Toc218862927)

[Computational performance and scalability 17](#_Toc218862928)

[**Processing time and multithreading effects** 17](#_Toc218862929)

[**Scalability with corpus size** 17](#_Toc218862930)

[**Memory usage** 17](#_Toc218862931)

[**Stability and practical considerations** 18](#_Toc218862932)

[**Table S1.** **Scalability and computational performance of HERMES for large-scale literature mining (4,459**) 18](#_Toc218862933)

[**Table S2.** **Scalability and computational performance of HERMES for medium-scale literature mining (371 articles**) 18](#_Toc218862934)

[Precision/Recall Assessment (ASE asymmetry in *C. elegans*) 19](#_Toc218862935)

[**Precision** 19](#_Toc218862936)

[**Recall** 19](#_Toc218862937)

[**Interpretation** 19](#_Toc218862938)

[**Table S3. Recall metrics for HERMES on ASE asymmetry in C. elegans** 20](#_Toc218862939)

[Precision/Recall Assessment (Cellulase production in *T. reesei*) 21](#_Toc218862940)

[**Precision** 21](#_Toc218862941)

[**Recall** 21](#_Toc218862942)

[**Interpretation** 21](#_Toc218862943)

[**Table S4. Recall metrics for HERMES on cellulase production in T. reesei** 22](#_Toc218862944)

[NER Validation Details 23](#_Toc218862945)

[**Definitions and metrics**. For each article and each entity class (gene names, proteins, diseases, chemicals, cells, organisms, tissues, pathways), HERMES recorded the set of extracted entity strings prior to validation (pre-validation) and the subset retained after validation (post-validation). For each entity class, we computed: 23](#_Toc218862946)

[**Validation rejection rates across entity classes** 23](#_Toc218862947)

[**Raw mentions vs. unique entities.** Pre-validation NER outputs consist of raw mention lists, in which the same entity string may appear repeatedly within a single article due to repetition across the main text, figure captions, tables, and supplementary materials. This duplication is especially pronounced for canonical regulators and neuron classes in C. elegans neurobiology. For example, in the top-ranked article (PMCID: 8735970), the gene symbol CHE-1 appears numerous times across multiple sections and captions. Prior to aggregation, HERMES deduplicates entity strings within each article, yielding a smaller set of unique pre-validation entities, which are then validated. The combined effects of deduplication and validation are summarized by distinguishing between raw mentions, deduplicated pre-validation entities, and final retained entities (Table S5). This distinction clarifies that a substantial portion of entity count reduction reflects consolidation of repeated mentions, rather than semantic filtering alone. 23](#_Toc218862948)

[**Gene-focused false positives generated prior to validation.** Pre-validation gene extraction intentionally favors sensitivity and therefore includes a substantial number of gene-like strings that do not correspond to biological gene entities. This behavior is illustrated clearly by the pre-validation gene list from PMCID: 8735970, excerpted below (abridged for clarity): 24](#_Toc218862949)

[**Gene-focused false negatives introduced by validation** 24](#_Toc218862950)

[**Interpretation and methodological implications.** Taken together, these results indicate that NER validation in HERMES performs two distinct but interrelated functions: 24](#_Toc218862951)

[**Table S5. Impact of deduplication and validation on extracted entity counts** 25](#_Toc218862952)

[Comparison of HERMES with LLM-based literature exploration tools 26](#_Toc218862953)

[**Functional scope and design philosophy** 26](#_Toc218862954)

[**Benchmarking against Elicit** 26](#_Toc218862955)

[**Benchmarking against Perplexity** 26](#_Toc218862956)

[**Overlap between tools** 26](#_Toc218862957)

[**Speed and throughput comparison** 27](#_Toc218862958)

[**Summary of trade-offs** 28](#_Toc218862959)

**Availability and requirement**

Project name: HERMES

Project home page: <https://github.com/julien-charest/hermes>

Operating system: Platform independent

Programming language: Python (3.11)

Dependencies: Python (3.11), BioPython, pandas, numpy, requests; BeautifulSoup4 and lxml for XML parsing; matplotlib for visualization; Tk for the GUI; fpdf2 for PDF report generation; PyTorch (CPU) and Hugging Face Transformers (tokenizers, sentencepiece, protobuf) for LLM-based summarization; spaCy and SciSpaCy (with the en_ner_bionlp13cg_md model) for biomedical named-entity recognition.

Other requirements: Internet connection for PMC, MyGene.info, MyDisease.info, MyChem.info, EBI OLS4 and MyPathway,info queries.

License: GPL-3

Any restriction to use by non-academics: none

# **Installation**

HERMES is distributed as an open-source Python application and is installed from its GitHub repository. Installation is performed using Conda to ensure consistent dependency management.

The source code is obtained by cloning the repository and navigating to the project directory using the following commands:

*git clone https://github.com/julien-charest/hermes.git
cd hermes*

A dedicated Conda environment is then created from the provided environment specification file and activated:

*conda env create -n hermes -f environment.yml
conda activate hermes*

By default, HERMES runs on CPU-only systems. On machines equipped with a compatible NVIDIA GPU (Linux or Windows) and appropriate drivers, GPU acceleration for LLM-based summarization can optionally be enabled by installing the CUDA-enabled PyTorch package:

*conda install pytorch-cuda=11.8 -c pytorch -c nvidia*

After installation, the application is launched from the project root directory using:

*python hermes.py*

# **Main script overview (hermes.py) and global configuration**

The main entry point of HERMES is the hermes.py script. This script (i) initializes global runtime settings, (ii) exposes user-configurable parameters that control retrieval scope and performance, (iii) launches the Tkinter-based graphical user interface (GUI), and (iv) orchestrates the end-to-end workflow by calling the dedicated pipeline modules for mining/parsing, scoring, summarization, visualization, and report generation. Several key settings can be adjusted directly in hermes.py prior to execution.

**NCBI Entrez access and retrieval scope.** HERMES queries PubMed Central (PMC) through the NCBI Entrez API. Users may optionally supply an NCBI API key by setting Entrez.api_key. Without an API key, Entrez requests are limited to approximately ~3 requests/second, whereas providing a valid key increases the rate limit to ~10 requests/second. The maximum number of PMC records retrieved per query is controlled by retmax (default: 100000), allowing users to either screen a large corpus or restrict retrieval for faster exploratory runs.

**Performance and parallelization.** To accelerate processing, HERMES parallelizes independent tasks using concurrent.futures.ThreadPoolExecutor. The number of worker threads used for (i) article mining/parsing and (ii) article summarization is controlled by max_workers_mining and max_workers_summary, respectively (default: 10 each). Increasing these values can reduce runtime until throughput becomes limited by external constraints (e.g., Entrez rate limits and network latency).

**Summarization configuration.** Summarization is invoked by hermes.py but configured within modules/summarizing.py, including the default LLM model selection and hardware settings (CPU/GPU). Users wishing to change summarization behavior (e.g., selecting a different Hugging Face model) should modify the settings in modules/summarizing.py (see module customization sections below).

After users provide query parameters through the GUI (request title, email address for Entrez identification, PMC query terms, scoring keywords, strict mode toggle, number of top results), hermes.py launches the main pipeline function (literature_miner). The workflow proceeds through: (1) PMC query and retrieval of PMCIDs, (2) multi-threaded mining/parsing of full-text XML records (with bounded retries and logging of failures), (3) scoring and ranking via the scoring module, (4) export of the full screened corpus as a CSV file (hermes_results.csv) for downstream analysis, (5) generation of summary figures, (6) concurrent LLM-based summarization of the top-ranked articles, and (7) compilation of a structured PDF report.

For each run, outputs are written to a user-selected destination folder under a run-specific directory containing (i) a log file capturing parameters and runtime information, (ii) summary figures, (iii) the full results table for the screened corpus, and (iv) the final PDF report. Module-level customization (mining/parsing, scoring, summarization, visualization, and PDF reporting) is described in the following Supplementary sections.

# **User-defined parameters (GUI inputs)**

HERMES is configured through a small set of user-defined parameters entered in the graphical user interface. These inputs control how results are retrieved, screened, ranked, and exported.

**Request title.** The request title is used to label the analysis and define the output directory and filenames. All outputs for a run (log file, figures, CSV results table, and PDF report) are saved under a run-specific folder named using the current date and the request title. Reusing the same request title on the same date may overwrite existing outputs in the selected destination directory.

**Email address (Entrez identification).** NCBI requires user identification for Entrez access. HERMES uses the email address to configure Entrez.email prior to querying PMC. If an NCBI API key is supplied in hermes.py, it should correspond to the same user account associated with the provided email to ensure proper rate-limit handling and compliance with NCBI usage policies.

**Query terms (PMC query).** Query terms define the PubMed Central search and are passed directly to the Entrez esearch call against the PMC database. Standard PubMed query syntax and filters are supported (e.g., field tags and filtering operators), enabling users to refine the retrieved corpus prior to screening and scoring.

**Scoring keywords.** Scoring keywords are provided as a comma-separated list and are used for full-text screening and ranking after retrieval. Keywords are matched against the parsed full-text content using **exact substring matching**. This approach is transparent and efficient but does not automatically expand synonyms or acronyms (e.g., “Caenorhabditis elegans” will not match “C. elegans” unless explicitly included). At the same time, substring matching can capture simple morphological extensions when the literal substring is present; for example, the keyword “toxic” will match occurrences such as “toxicity” or “toxicology”. Users aiming for higher recall are therefore encouraged to include common variants and alternative spellings as additional keywords.

**Strict mode.** When strict mode is enabled, HERMES applies a deterministic inclusion rule during screening: any article that does not contain **all** user-defined keywords is discarded from ranking and excluded from downstream reporting. This supports high-specificity (“negative”) screening use cases where co-occurrence of multiple terms is required.

**Number of results (report size).** The number of results defines how many top-ranked articles are included in the final PDF report with automated summaries and entity extraction. Importantly, the **entire retrieved corpus** is still mined, screened, scored, and exported to disk as a CSV table; the report size only controls the subset of highest-scoring articles that receive the most detailed per-article processing and are included in the PDF.

# **Mining module (modules/mining.py)**

The mining module is responsible for retrieving full-text PMC records and extracting the core metadata and text fields used downstream for scoring, summarization, visualization, and reporting. The primary entry point is mine_pmcid(pmcid, keywords), which is executed in parallel across PMCIDs by the main pipeline.

**Record retrieval (fetch_record).** For each PMCID, HERMES downloads the full PMC XML record using the Entrez efetch endpoint (db="pmc", retmode="xml", rettype="full"). To comply with NCBI usage policies, requests are rate-limited using randomized delays. When an NCBI API key is configured (Entrez.api_key), shorter delays are used to reflect the higher allowed request rate; otherwise, more conservative delays are applied.

**XML parsing and metadata extraction (mine_pmcid).** Retrieved XML is parsed with BeautifulSoup using the lxml parser. The module extracts bibliographic metadata including article title, journal title, publication year, author list, DOI (when available), and PMC-provided keyword tags. The abstract is extracted when present, with fallbacks for records with missing or irregular abstract markup.

**Citation count retrieval (get_citations).** When a PubMed identifier (PMID) is available, the module queries the Entrez elink endpoint (linkname="pubmed_pubmed_citedin") to estimate citation count as the number of “cited-in” links returned for the PMID. This step follows the same rate-limiting logic as XML retrieval and includes bounded retries to handle transient failures.

**Full-text extraction and keyword statistics.** To approximate the main article body, the module extracts the text region between the Introduction and References section headers when present, converts it to lower case, and computes total word count. Keyword frequencies are then computed using exact substring counting (text.count) for each user-provided scoring keyword. If a usable main-text region cannot be identified (e.g., word count is zero), keyword frequencies are computed on the abstract as a fallback.

**Returned data structure.** For each processed PMCID, mine_pmcid returns a dictionary containing identifiers (PMCID/PMID), bibliographic fields (title, journal, year, authors, DOI), PMC keyword tags, citation count, keyword occurrence counts, word count, and the abstract. These per-article dictionaries are aggregated into a pandas DataFrame by the main script and subsequently passed to the scoring, summarization, visualization, and reporting modules.

**Customization.** Users wishing to customize mining behavior may adjust (i) how the main-text region is defined (e.g., alternative section boundaries or inclusion of full XML text), (ii) the keyword counting strategy (e.g., token-boundary matching or regular expressions), and/or (iii) the pacing/rate-limiting logic for Entrez requests.

# **Scoring module (modules/scoring.py)**

HERMES ranks retrieved articles using a composite relevance score that integrates textual relevance, citation impact, and publication age in a transparent and fully customizable manner. Scoring is performed after mining the complete PMC corpus matching a given query and operates on the full text of each article.

**Term frequency and inverse document frequency.** For each article, term frequency (TF) values are computed for all user-defined scoring keywords as the number of occurrences of a keyword normalized by the total word count of the article body. This normalization prevents longer articles from being systematically favored due to higher absolute keyword counts. Across the full retrieved corpus, inverse document frequency (IDF) values are computed for each keyword as:

IDF(term) = log(N / df_term)

where *N* is the total number of retrieved articles and *df_term* is the number of articles in which the keyword occurs at least once. This formulation downweights ubiquitous keywords and increases the contribution of terms specific to a subset of articles. The TF–IDF contribution for a given article is obtained by summing the product of TF and IDF values across all scoring keywords present in the document.

**Composite relevance score.** The final relevance score for each article is computed as:

Score = (w_tf_idf × TFIDF_sum) × (1 + w_cit × Citations) / (1 + w_year × Age)

where *Citations* is the number of PubMed citations associated with the article and *Age* is the difference between the current year and the year of publication. This formulation ensures that textual relevance remains the dominant driver of ranking while allowing citation impact and recency to modulate scores in a controlled manner.

**Effect of weighting parameters.** The influence of each scoring component is governed by explicit weighting parameters:

- **w_tf_idf (text relevance weight).** Controls the linear contribution of TF–IDF–based semantic relevance. Increasing w_tf_idf amplifies differences between articles driven by keyword specificity and frequency, whereas decreasing it compresses score differences between articles with similar TF–IDF profiles. Setting w_tf_idf = 0 disables text-based ranking entirely and results in all scores being zero.
- **w_cit (citation boost weight).** Modulates the multiplicative contribution of citation counts via the term (1 + w_cit × Citations). Increasing w_cit progressively favors highly cited papers while preserving TF–IDF–based ordering. Setting w_cit = 0 removes citation influence completely, yielding a score driven solely by text relevance and publication age.
- **w_year (age penalty weight).** Controls the penalty applied to older publications through division by (1 + w_year × Age). Larger values increasingly down-weight older articles relative to recent publications. Setting w_year = 0 fully disables any recency bias, resulting in age-invariant ranking. A linear penalty was chosen to avoid hard cutoffs or exponential decay that would disproportionately suppress older but semantically relevant or seminal work.

Because the three components are multiplicatively combined, high textual relevance is a necessary condition for high ranking, while citation impact and recency act as secondary modifiers. All weights are user-adjustable at the code level, enabling transparent customization of ranking behavior without opaque heuristics.

**Strict mode filtering.** In addition to continuous scoring, HERMES supports an optional strict mode for targeted or negative literature screening. In strict mode, the same scoring function is applied, but articles missing any user-defined scoring keyword are discarded by an explicit conditional rule prior to ranking. This allows users to exclude, at corpus scale, any articles that do not simultaneously mention all required terms (e.g., enforcing co-occurrence of multiple genes or concepts), without introducing a separate scoring formulation.

# **Summarizing module (modules/summarizing.py)**

The summarization module generates the per-article “deep dive” content included in the final PDF report for the user-selected top-ranked articles. For each selected PMCID, the module (i) retrieves the full PMC XML record, (ii) preprocesses and segments the article into narrative sections, (iii) generates LLM-based summaries, (iv) extracts figure titles/captions, and (v) performs biomedical named-entity recognition (NER) followed by lightweight validation against external resources to reduce false positives. The primary entry point is summarize_article(pmcid), which is executed concurrently by the main script.

**Model configuration and compute device.** Summarization is implemented using a Hugging Face summarization pipeline built on a T5-family model. The default model is set by LLM_MODEL_NAME (default: google/flan-t5-large) and can be replaced with any Hugging Face–compatible summarization model depending on user requirements and available computational resources. Execution hardware is controlled by the device parameter: device = -1 runs on CPU, while device = 0 uses the first CUDA-enabled GPU. When a GPU is available, LLM inference can be substantially accelerated. Biomedical NER is performed using spaCy/SciSpaCy with the model specified by NER_MODEL_NAME (default: en_ner_bionlp13cg_md). Both summarization and NER models are loaded once at module import to avoid repeated initialization overhead during batch processing.

**XML retrieval and preprocessing.** For each PMCID, the module retrieves the full PMC XML record via Entrez (fetch_record_xml). Requests are rate-limited using randomized delays, with shorter delays enabled when an NCBI API key is configured (Entrez.api_key). The XML is parsed with BeautifulSoup (lxml-xml parser). Prior to summarization, the module removes content that tends to degrade summary quality or inflate input length, including tables (<table-wrap>, <table>), reference markers and reference blocks (<xref>, <ref>), and supplementary material sections. Inline formatting tags (e.g., italic/bold/sup/sub) are unwrapped to produce cleaner plain text while preserving scientific characters and notation.

**Section extraction and summary generation.** Articles are split into structured sections based on <title> headings (extract_sections). To focus on narrative content, HERMES filters extracted sections to a set of common “core” section types (e.g., Introduction/Background, Results, Discussion, Conclusion). Each retained section is cleaned (clean_text) and summarized using an instruction-style prompt (“Summarize the following scientific text in 3–6 sentences…”) in a batched call to the Hugging Face pipeline (summarize_sections). Section summaries are returned as labeled blocks and concatenated for inclusion in the report. Summarization behavior can be adjusted via parameters such as min_new_tokens, max_new_tokens, and batch_size.

**Figure caption extraction.** Figure titles/captions are extracted separately from <fig> elements (get_figures) to provide additional context in the report. The module preferentially extracts an explicit <title> within figure captions when present, otherwise falling back to the first caption paragraph.

**Named-entity recognition and validation.** After preprocessing, the module runs biomedical NER over the cleaned article text using SciSpaCy. Entity candidates are extracted by label (genes/gene products, proteins, diseases, chemicals, cells, organisms, tissues, pathways). To reduce false positives, each candidate surface form is validated using entity-type–specific resolvers and ontologies via lightweight exact (case-insensitive) matching rules (e.g., MyGene.info for gene symbols and UniProt accessions, MyDisease.info/MyChem.info/MyPathway.info, and EBI OLS4 for CL/CLO, UBERON/BTO/FMA, and NCBITaxon). The module reports deduplicated validated surface forms per entity type; if no validated entities are found for a category, “N/A” is reported.

**Returned output structure.** summarize_article(pmcid) returns a structured result object containing the combined summary text, validated entity lists for each category, extracted figure text, and section title metadata. These outputs are merged back into the main results table and rendered in the final PDF report.

**Customization points.** Users can customize summarization and enrichment behavior by modifying: (i) LLM_MODEL_NAME (choice of Hugging Face model) and device (CPU/GPU selection), (ii) section filtering rules (allowed_keys) to include or exclude additional manuscript sections, (iii) summarization hyperparameters (token limits, batch size, decoding options), and (iv) validation logic and resources used for entity filtering.

# **Visualization module (modules/generating_graphs.py)**

The visualization module generates standardized summary figures that provide a compact overview of the retrieved corpus and the scoring outcome. All plots are created with matplotlib and written to the run-specific figures directory. The module is called after scoring is completed, ensuring that figures summarize both corpus metadata and ranking-related statistics.

**Publication-year distribution.** graph_pub_years(dataframe, request_id, dir) generates a line plot showing the number of retrieved articles per publication year. Articles are grouped by the Year field and counted using the number of PubMed IDs per year. The resulting figure provides a high-level view of temporal coverage and potential publication trends in the retrieved corpus.

**Associated keyword frequency.** graph_associated_keywords(dataframe, request_id, dir) generates a bar chart of the 25 most frequent PMC-associated keywords extracted during XML parsing. Keyword lists are flattened across all articles, aggregated, and ranked by the number of articles in which each keyword appears. This figure summarizes the topical composition of the retrieved set using author-/publisher-provided keyword annotations.

**Summary statistics overview.** graph_stats_summary(dataframe, request_id, dir) generates a multi-panel summary figure containing three boxplot-based visualizations: (i) citation counts per article, (ii) per-keyword occurrence counts across articles (one boxplot per scoring keyword), and (iii) final relevance scores. Each subplot overlays scatter points on the boxplots to visualize distribution shape and outliers. Together, these panels provide a compact quality-control view of how citation metadata, keyword matching, and composite scoring are distributed across the screened corpus.

**Customization.** Users may customize visualization behavior by editing this module, including: (i) the set of figures produced, (ii) the number of displayed associated keywords (default top 25), (iii) plot aesthetics (e.g., titles, axis formatting, or color palettes), and (iv) the inclusion of additional corpus-level metrics (e.g., TF–IDF distributions, runtime diagnostics, or per-section summary statistics).

# **Report generation module (modules/generating_report.py)**

The report generation module compiles mining, scoring, visualization, and enrichment outputs into a structured PDF document intended for rapid human review of the retrieved literature. The primary entry point is generate_pdf_report(...), which receives run metadata, the scored results table, pre-generated figures, and TF–IDF statistics, and writes a self-contained PDF report to the run output directory.

**Report structure and layout.** PDF generation is implemented using fpdf2. A custom PDF_Report class defines a standardized page layout, including a header (report title, run date, and optional logo) and a footer with page numbering. The header includes error handling so the report can still be produced if optional branding assets (e.g., the logo file) cannot be loaded. The report is organized into three main parts:

1. **Overview page (query summary).** The first page summarizes the query context and run statistics, including the query date, query terms, scoring keyword set, execution mode (default vs strict), the number of successfully parsed articles relative to total hits, and the IDF values (“keyword rarity”) computed for the scoring keywords. Two overview figures generated by the visualization module are embedded: the publication-year distribution and the top associated PMC keyword frequencies.
2. **Corpus-level statistics and ranked results table.** The second page embeds a summary statistics figure (citations, keyword count distributions, score distribution) and presents a tabular overview of the ranked results. Each row reports publication year, journal, first author (or “et al.”), truncated title, citation count, and the final relevance score. The report includes direct hyperlinks to the corresponding article landing pages via the DOI resolver (when a DOI is available), enabling one-click navigation from the PDF to the primary source.
3. **Per-article detailed pages (top-ranked subset).** For each top-ranked article included in the report, HERMES generates a dedicated page containing: bibliographic metadata (title, authors, journal, year, PMCID/PMID, DOI), citation count, relevance score, raw keyword hit counts and normalized TF values, the abstract, LLM-generated summaries (section-wise blocks), extracted figure titles/captions, and validated biomedical entity lists (genes, proteins, diseases, chemicals, cells, organisms, tissues, pathways). These per-article pages are designed to allow rapid triage of whether a search yielded scientifically useful hits.

**Robustness and output writing.** To reduce failure modes during file output, the module writes the final PDF with a small retry loop and checks that the generated file exists and has non-zero size before reporting success. Warnings and failures encountered during report assembly (e.g., missing logo assets or write errors) are appended to the run log file.

**Customization.** Users wishing to customize reporting can modify this module to adjust: (i) which figures and statistics are embedded, (ii) table columns and truncation lengths, (iii) the level of per-article detail included, (iv) hyperlink behavior (e.g., PMID/PMCID vs DOI links), and (v) overall page layout (fonts, margins, headers/footers).

# **Utility and helper functions module (modules/adding_functions.py)**

The adding_functions module provides a small collection of utility functions that support file handling, resource management, and safe text output across the HERMES pipeline. While it does not implement core mining or analysis logic, it plays a critical role in ensuring portability, reproducibility, and robustness of file and report generation.

**Resource path resolution.** The function ressource_path(relative_path) resolves paths to bundled application resources (e.g., logos or static assets). It is designed to work both in standard Python execution environments and in packaged/distributed contexts (e.g., PyInstaller builds), by checking for the presence of the _MEIPASS attribute. This ensures that HERMES can reliably locate auxiliary files regardless of how it is deployed.

**Output directory management.** The function create_reports_folder(working_directory, request_id, date) creates the standardized directory structure used for each HERMES run. Given a user-selected working directory, a request identifier, and the run date, it initializes:

- a top-level report directory named <date>_<request_id>,
- a figures/ subdirectory for all generated plots,
- a results/ subdirectory for tabular outputs (e.g., CSV files).

This centralized function ensures consistent naming and output layout across runs, facilitating reproducibility, downstream automation, and comparison of multiple queries.

**String sanitization for safe output.** The function filter_string(string) removes unsupported or non-ASCII characters from text before it is written to logs, filenames, or the PDF report. This prevents encoding-related errors in fpdf2, avoids malformed file outputs, and ensures compatibility across operating systems and PDF viewers. The function retains alphanumeric characters and a restricted set of common punctuation symbols sufficient for scientific text.

**Role within the HERMES architecture.** Although lightweight, this module underpins several user-facing features of HERMES, including robust report generation, portable resource handling, and clean presentation of metadata and summaries. Users who wish to extend HERMES (e.g., adding alternative output formats or additional resource files) can adapt these helper functions to accommodate new workflows while preserving the overall structure of the pipeline.

# **Output directory structure and generated results**

For each HERMES run, results are written to a self-contained and reproducible directory structure that captures both the complete screening output and the curated, user-facing report. The base output directory is created at the user-selected location and named using the query date and request title (<YYYY-MM-DD>_<request_id>), ensuring that multiple runs can be archived and compared unambiguously.

**Top-level report directory.** The root directory of a HERMES run contains:

- **Final PDF report** (<date>_<request_id>.pdf): a comprehensive, human-readable report summarizing the query, scoring parameters, global statistics, and detailed per-article results. The report includes hyperlinks to external resources (e.g., DOI links), allowing rapid navigation to the original publications.
- **Log file** (<request_id>_log.txt): a plain-text execution log capturing query parameters, runtime status messages, warnings, errors, and performance summaries (e.g., processing time and peak memory usage). This file supports transparency and troubleshooting.
- **Subdirectories** (figures/ and results/), described below.

**results/ directory.** The results/ subdirectory contains machine-readable outputs intended for downstream analysis and reproducibility:

- **hermes_results.csv**: a complete table of all successfully parsed articles retrieved from PubMed Central, not only the top-ranked subset included in the PDF report. For each article, the table includes bibliographic metadata, citation counts, raw keyword counts, normalized term frequencies (TF), inverse document frequency (IDF)–weighted scores, and the final relevance score. This file enables users to re-rank articles, apply alternative thresholds, or integrate HERMES outputs into custom analytical pipelines without re-running the mining step.

**figures/ directory.** The figures/ subdirectory stores all graphical outputs generated during the analysis, in standard image formats suitable for reuse in presentations or manuscripts:

- **Publication year distribution** (*_pubyears.png): a line plot showing the number of retrieved articles per publication year.
- **Associated keyword frequencies** (*_asskeywords.png): a bar chart of the most frequent author-provided or indexed keywords associated with the retrieved corpus.
- **Statistical summary figure** (*_stats_summary.png): a composite figure summarizing citation counts, keyword frequencies, and relevance score distributions across the screened corpus.

**Design rationale.** This structured output layout separates human-readable summaries from machine-readable data while preserving both. By saving the full scored corpus to disk, HERMES supports transparent auditing of ranking decisions, reproducible research workflows, and flexible downstream reuse, beyond the immediate PDF report generated for interactive inspection.

# **Computational performance and scalability**

All performance benchmarks were conducted on a workstation running **Microsoft Windows 11 Pro (build 26200)** equipped with an **AMD Ryzen Threadripper Pro 5975WX** processor (32 physical cores, 63 logical processors, base clock 3.6 GHz), **512 GB RAM**, and an **NVIDIA GeForce RTX 4090 GPU**.

Performance was evaluated on two representative demonstration queries differing substantially in corpus size: (i) “cellulase production trichoderma reesei” (4,459 PubMed Central articles), and (ii) “ase asymmetry c elegans” (371 PubMed Central articles). For each query, HERMES was executed using **1, 10, and 20 concurrent worker threads**, corresponding to the max_workers_mining and max_workers_summary parameters.

**Processing time and multithreading effects.** For the larger corpus (T. reesei, 4,459 articles), **the literature mining stage** (full-text retrieval, parsing, and keyword counting) showed substantial gains from multithreading. Mining time decreased from **7,543 s** with a single worker to **1,936 s** with 10 workers (≈**3.9-fold speedup**), while increasing the worker count to 20 did not yield further improvements (**2,124 s**), indicating saturation by external I/O constraints. The **summarization stage** (LLM-based summarization of the top 25 ranked articles) also benefited from moderate concurrency, decreasing from **2,265 s** (1 worker) to **747 s** (10 workers), but showed diminished returns at higher concurrency (**992 s** at 20 workers), reflecting both Entrez fetch limits and model execution overhead. Consequently, **total wall-clock runtime** decreased from **9,826 s (163.8 min)** with one worker to **2,700 s (45.0 min)** with 10 workers, before increasing again to **3,134 s (52.2 min)** at 20 workers. A similar pattern was observed for the smaller corpus (C. elegans, 371 articles). **Mining time** decreased from **690 s** with one worker to **171 s** with 10 workers (≈**4.0-fold speedup**), with no further improvement at 20 workers (**183 s**). The **summarization stage** remained the dominant contributor to runtime, decreasing from **2,692 s** (1 worker) to **852 s** (10 workers), but increasing again to **1,095 s** at 20 workers. As a result, **total runtime** was reduced from **3,385 s (56.4 min)** with one worker to **1,026 s (17.1 min)** with 10 workers, and increased to **1,281 s (21.3 min)** at 20 workers. Across both corpus sizes, these results demonstrate that HERMES benefits strongly from **moderate multithreading**, with optimal performance observed at approximately **10 concurrent workers** under default NCBI Entrez access conditions. Beyond this point, **NCBI request rate limits and network I/O** dominate performance, leading to diminishing returns or instability at higher concurrency levels.

**Scalability with corpus size.** Mining time scaled approximately linearly with the number of retrieved articles. With a single worker, processing **4,459 articles required ~7,543 s**, whereas **371 articles required ~690 s**, corresponding to an ~11-fold increase in corpus size and a ~10.9-fold increase in mining time. This near-linear scaling reflects the per-article retrieval, parsing, and keyword counting strategy employed by HERMES and confirms predictable performance behavior as corpus size increases (Tables S1 & S2).

**Memory usage.** Peak resident memory usage increased with the number of concurrent workers. For both queries, peak memory usage was approximately **5.7 GiB** with one worker, **9.6–9.8 GiB** with 10 workers, and **12.5–13.1 GiB** with 20 workers. The increased memory footprint at higher concurrency reflects a larger number of in-flight article records and parallel summarization tasks. Given the test system's memory capacity, it was not a limiting factor in any benchmarked configuration (Tables S1 & S2).

**Stability and practical considerations.** At higher concurrency (20 workers), transient Entrez fetch errors were observed during the summarization stage for a subset of articles. These errors were handled by HERMES’s bounded retry logic, and all benchmark runs successfully completed summarization for the top-ranked articles. The results suggest that **moderate multithreading (≈10 workers)** offers the best trade-off between speed, stability, and resource usage under typical NCBI access constraints.

Overall, these benchmarks demonstrate that HERMES scales efficiently with corpus size, achieves substantial speedups through multithreading, and remains practical on standard high-end desktop or workstation hardware without requiring specialized infrastructure.

### **Table S1.** **Scalability and computational performance of HERMES for large-scale literature mining (4,459***)*

| **Workers** | **Mining stage (s)** | **Mining (min)** | **Summarize top 25 (s)** | **Summarize (min)** | **Total runtime (s)** | **Total (min)** | **Peak RSS (MiB)** |
| --- | --- | --- | --- | --- | --- | --- | --- |
| 1 | 7543.45 | 125.72 | 2265.02 | 37.75 | 9825.98 | 163.77 | 5871.2 |
| 10 | 1935.81 | 32.26 | 746.71 | 12.45 | 2700.14 | 45.00 | 9641.0 |
| 20 | 2123.89 | 35.40 | 992.08 | 16.53 | 3133.98 | 52.23 | 12484.5 |

### **Table S2.** **Scalability and computational performance of HERMES for medium-scale literature mining (371 articles***)*

| **Workers** | **Mining stage (s)** | **Mining (min)** | **Summarize top 25 (s)** | **Summarize (min)** | **Total runtime (s)** | **Total (min)** | **Peak RSS (MiB)** |
| --- | --- | --- | --- | --- | --- | --- | --- |
| 1 | 690.21 | 11.50 | 2691.85 | 44.86 | 3384.75 | 56.41 | 5722.5 |
| 10 | 171.27 | 2.85 | 851.65 | 14.19 | 1026.04 | 17.10 | 9788.0 |
| 20 | 182.83 | 3.05 | 1095.04 | 18.25 | 1280.75 | 21.35 | 13073.3 |

# **Precision/Recall Assessment (ASE asymmetry in *C. elegans*)**

To assess retrieval quality for a well-characterized biological topic, we evaluated HERMES on the query “ASE asymmetry *C. elegans*” using both default and strict modes, with keywords “ASE, *lsy-6*, *che-1*”. Precision and recall were estimated against an expert-curated reference set and expert judgment, acknowledging the structural limitations imposed by full-text availability in PubMed Central (PMC).

**Precision.** Precision was evaluated by **manual expert assessment** rather than automated labeling, reflecting the absence of a comprehensive negative reference set. A co-author with domain expertise in ASE neuronal asymmetry independently evaluated the relevance of retrieved articles. **Default mode (top-25 results):** **9 of 24 unique articles** were judged as highly relevant to ASE asymmetry. One entry appeared twice due to **preprint (bioRxiv) and published (eLife) versions of the same study**, yielding **9/24 = 37.5% precision** after deduplication. **Strict mode (top-10 results): 9 of 10 articles** were judged as highly relevant, corresponding to **90% precision**. These findings demonstrate that strict keyword enforcement substantially improves precision by filtering tangential literature, at the cost of reduced breadth.

**Recall.** The gold/reference set was derived from the primary citations of Charest et al., 2020, supplemented by Charest et al., 2020 itself, yielding 12 canonical publications spanning foundational discovery, regulatory mechanisms, and maintenance of ASE left/right asymmetry. This citation-based set reflects expert consensus on core literature rather than an exhaustively enumerated ground truth. Importantly, only two of these gold-set publications permit automated full-text XML retrieval via Entrez (PMC3116913; PMC7704111). Several others possess PMC identifiers but are subject to publisher restrictions that prevent XML download, while the remainder are not deposited in PMC. As HERMES operates exclusively on retrievable full-text PMC XML, recall estimates must therefore be interpreted in light of this eligibility constraint. **Absolute recall:** HERMES retrieved **2 of 12** gold-set papers in its ranked outputs (16.7%). **Eligible recall (relative to PMC-retrievable gold subset).** Of the **2 gold-set papers with Entrez-retrievable full text**, **both were successfully retrieved**, corresponding to an **eligible recall of 100%**. These results indicate that recall is primarily limited by **publisher access restrictions rather than retrieval or scoring failures**, and that HERMES reliably recovers gold-standard literature when full-text access is permitted (Table S3).

**Interpretation.** Taken together, these results illustrate a clear trade-off between **precision and inclusivity**: **Default mode** favors broader discovery and contextual expansion but includes peripheral studies. **Strict mode** produces a compact, high-precision set closely aligned with expert expectations. Crucially, recall limitations are dominated by **systemic constraints in full-text availability**, not by deficiencies in HERMES’ mining, scoring, or ranking strategy. When evaluated against technically accessible literature, HERMES achieves complete recovery of gold-standard references while offering transparent, reproducible scoring.

### **Table S3. Recall metrics for HERMES on ASE asymmetry in C. elegans**

| **#** | **Gold reference** | **PMID** | **PMCID** | **Full-text XML retrievable via Entrez?** | **In HERMES default top-25?** | **In HERMES strict?** |
| --- | --- | --- | --- | --- | --- | --- |
| 1 | Cochella & Hobert 2012 (Cell) | 23201143 | 3529140 | No (publisher restriction) | No | No |
| 2 | Etchberger et al. 2009 (Development) | 19060335 | 2685964 | No (publisher restriction) | No | No |
| 3 | Etchberger et al. 2007 (Genes Dev.) | 17606643 | 1899474 | No (publisher restriction) | No | No |
| 4 | Good et al. 2004 (Development) | 15056620 | — | No PMCID / not in PMC | No | No |
| 5 | Hobert 2014 (Genesis) | 24510690 | — | No PMCID / not in PMC | No | No |
| 6 | Johnston & Hobert 2005 (Development) | 16291785 | — | No PMCID / not in PMC | No | No |
| 7 | Johnston & Hobert 2003 (Nature) | 14685240 | — | No PMCID / not in PMC | No | No |
| 8 | Poole et al. 2011 (PLoS Genet.) | 21698137 | 3116913 | Yes | Yes | Yes |
| 9 | Poole & Hobert 2006 (Curr. Biol.) | 17141609 | — | No PMCID / not in PMC | No | No |
| 10 | Sarin et al. 2007 (Genetics) | 17717195 | 1950618 | No (publisher restriction) | No | No |
| 11 | Suzuki et al. 2008 (Nature) | 18596810 | 2984562 | No (publisher restriction) | No | No |
| 12 | Charest et al. 2020 (Developmental Cell) | 33002421 | 7704111 | Yes | Yes | Yes |

# **Precision/Recall Assessment (Cellulase production in *T. reesei*)**

To assess retrieval quality for a second, mechanistically rich and extensively studied biological topic, we evaluated HERMES on the query “cellulase production *trichoderma reesei*” with the keywords “cellulase, *reesei*, *cbh1*, *xyr1*”, using both default and strict mode. Precision and recall were estimated against an expert-curated reference set and expert judgment, while explicitly accounting for structural constraints imposed by full-text availability in PubMed Central (PMC).

**Precision.** Precision was evaluated by **manual expert assessment** rather than automated labelling, reflecting the absence of a comprehensive negative reference set and the heterogeneous scope of the cellulase literature. A co-author with domain expertise in cellulase production independently evaluated the relevance of retrieved articles. Retrieved articles were assessed for direct relevance to transcriptional regulation of cellulase expression in *T. reesei*, with emphasis on xyr1, cbh1, promoter architecture, chromatin regulation, and regulatory network components. **Default mode (top-25 results):** **of the 25 retrieved articles, 20 were judged to be highly relevant, corresponding to a precision of 80% (20/25). The remaining entries primarily reflected broader fungal biotechnology, enzyme engineering, or peripheral regulatory pathways.** **Strict mode (top-25 results): in strict mode, 19 of 25 retrieved articles were judged as highly relevant, yielding a precision of 76% (19/25). Although strict filtering marginally reduced absolute precision, it produced a more homogeneous set centered on core transcriptional regulators and promoter-level mechanisms. Together, these results indicate that both modes achieve high precision for this topic, with strict mode emphasizing mechanistic specificity and default mode providing slightly broader contextual coverage.**

**Recall.** The gold/reference set consisted of 15 expert-selected publications spanning promoter chromatin regulation, transcription factor structure and function, regulatory networks, and recent engineering strategies, describing the current state of knowledge regarding the regulation of cbh1 expression by Xyr1. This set includes both primary research articles and authoritative reviews and reflects expert consensus rather than an exhaustively enumerated ground truth. Importantly, 14 of the 15 gold-set publications permitted automated full-text XML retrieval via Entrez, while one recent review published in a non-PMC journal is not technically accessible by HERMES. **Absolute recall:** HERMES retrieved **10 of 15** gold-standard papers in its default mode-ranked outputs (66.7%). In strict mode, HERMES retrieved 11 of 15 gold-standard papers, yielding an absolute recall of 73.3%. **Eligible recall (relative to PMC-retrievable gold subset).** Of the **14 gold-standard papers with Entrez-retrievable full text**, **10 were successfully retrieved in default mode**, yielding an **eligible recall of 71.4%**. Of the **14 gold-set papers with Entrez-retrievable full text**, **11 were successfully retrieved in strict mode**, corresponding to an **eligible recall of 78.6%**. Notably, one mechanistically important study (Mello-de-Sousa et al., 2016) was not retrieved despite in full textbeing available availability, reflecting competition for ranking rather than technical access limitations (Table S4).

**Interpretation.** Taken together, these results demonstrate that HERMES performs robustly on a complex, regulator-dense query in fungal biology. Precision remains high in both modes, while recall is primarily influenced by ranking depth and corpus competition rather than XML availability constraints. Unlike the ASE asymmetry case, where recall was dominated by publisher restrictions, most canonical cellulase studies are technically accessible, allowing a more direct evaluation of retrieval behavior. Strict mode modestly improves recall relative to the gold set while maintaining high precision, whereas default mode favors broader contextual inclusion. Overall, HERMES reliably recovers most of the expert-recognized core literature for cellulase regulation in *T. reesei*, with transparent limitations driven by ranking scope rather than systemic access barriers.

### **Table S4. Recall metrics for HERMES on cellulase production in T. reesei**

| **#** | **Gold reference** | **PMID** | **PMCID** | **Full-text XML retrievable via Entrez?** | **In HERMES default top-25?** | **In HERMES strict top-25?** |
| --- | --- | --- | --- | --- | --- | --- |
| 1 | Mello-de-Sousa et al. 2016 (*Biotechnol Biofuels*) | 27363706 | 4922684 | Yes | No | No |
| 2 | Zhao et al. 2023 (*Synth Syst Biotechnol*) | 38187093 | 10770280 | Yes | No | Yes |
| 3 | Yan et al. 2021 (*Biotechnol Biofuels*) | 38650205 | 10991602 | Yes | Yes | No |
| 4 | Mattam et al. 2022 (*Microbial Cell*) | 35317826 | 8939176 | Yes | Yes | Yes |
| 5 | Adnan et al. 2022 (*Microbio Res*) | **35339938** | — | No PMCID / not in PMC | No | No |
| 6 | Lichius et al. 2015 (*BMC Genomics*) | 25909478 | 4409711 | Yes | Yes | Yes |
| 7 | Zheng et al. 2020 (*PLoS Genet*) | 32877410 | 7467262 | Yes | Yes | Yes |
| 8 | Shen et al. 2022 (*Biotechnol Biofuels*) | 36528622 | 9759857 | Yes | Yes | Yes |
| 9 | Arai et al. 2022 (*Sci Rep*) | 36376415 | 9663580 | Yes | Yes | Yes |
| 10 | Wang et al. 2021 (*PLoS Genet*) | 33606681 | 7894907 | Yes | Yes | Yes |
| 11 | Jiang et al. 2020 (*Front Microbiol*) | 32765463 | 7381231 | Yes | Yes | Yes |
| 12 | Han et al. 2020 (*Synth Syst Biotechnol*) | 32695894 | 7365963 | Yes | No | Yes |
| 13 | Liu et al. 2019 (*Microb Cell Fact*) | 31077201 | 6509817 | Yes | No | Yes |
| 14 | Amore et al. 2013 (*Curr Genomics*) | 24294104 | 3731814 | Yes | Yes | Yes |
| 15 | Seiboth et al. 2012 (*Mol Microbiol*) | 22554051 | 3370264 | Yes | Yes | No |

# **NER Validation Details**

Named-entity recognition (NER) in HERMES is intended to support exploratory synthesis of full-text biomedical literature rather than to maximize recall under a single gold-standard ontology. To this end, HERMES employs a two-stage entity handling strategy: (i) permissive extraction using general biomedical NER models to capture a broad set of candidate entities, followed by (ii) deterministic, class-specific validation and normalization to reduce spurious outputs and improve interpretability. Validation comprises rule-based filtering in combination with normalization and identifier resolution against external reference databases, consolidation of surface-form variants, and within-article deduplication of repeated mentions.This section provides a quantitative and qualitative assessment of the validation step, documenting its impact on entity counts, the nature of pre-validation false positives, and the introduction of validation-induced false negatives. All analyses are based on a representative example query (“ASE asymmetry *C. elegans*”, with keywords “ASE, *che-1*, *lsy-6*”), comprising 25 full-text articles retrieved from PubMed Central.

**Definitions and metrics**. For each article and each entity class (gene names, proteins, diseases, chemicals, cells, organisms, tissues, pathways), HERMES recorded the set of extracted entity strings prior to validation (pre-validation) and the subset retained after validation (post-validation). For each entity class, we computed:

- **N_pre**: number of unique entity strings extracted prior to validation
- **N_post**: number of unique entity strings retained after validation
- **N_removed =** N_pre − N_post

We report the validation rejection rate, defined as:

$$\text{Validation rejection rate}=\frac{N_{removed}}{N_{pre}}$$

**Validation rejection rates across entity classes**. Across all detected entity classes in this dataset (genes, cells, organisms, tissues), validation reduced the total number of unique extracted entities from 4,448 pre-validation to 950 post-validation, corresponding to an overall micro-averaged rejection rate of 78.6%. Rejection rates varied markedly by entity class, with gene names exhibiting a lower, but still substantial, rejection rate, and cell and organism mentions showing consistently higher rejection rates. Entity classes with no pre-validation detections in this dataset (proteins, diseases, chemicals, pathways) were excluded from rate calculations.

**Raw mentions vs. unique entities.** Pre-validation NER outputs consist of raw mention lists, in which the same entity string may appear repeatedly within a single article due to repetition across the main text, figure captions, tables, and supplementary materials. This duplication is especially pronounced for canonical regulators and neuron classes in *C. elegans* neurobiology. For example, in the top-ranked article (PMCID: 8735970), the gene symbol CHE-1 appears numerous times across multiple sections and captions. Prior to aggregation, HERMES deduplicates entity strings within each article, yielding a smaller set of unique pre-validation entities, which are then validated. The combined effects of deduplication and validation are summarized by distinguishing between raw mentions, deduplicated pre-validation entities, and final retained entities (Table S5). This distinction clarifies that a substantial portion of entity count reduction reflects consolidation of repeated mentions, rather than semantic filtering alone.

**Gene-focused false positives generated prior to validation.** Pre-validation gene extraction intentionally favors sensitivity and therefore includes a substantial number of gene-like strings that do not correspond to biological gene entities. This behavior is illustrated clearly by the pre-validation gene list from PMCID: 8735970, excerpted below (abridged for clarity):

*Examples from pre-validation gene_names (PMCID 8735970):* BurghtServaas Nhttps://orcid.org/0000-0002, WalczakAleksandra MSenior, BYAuthor, ASE, ASE neuron, ASE-specific, ASER, ADF, ASK, AWC, auxin, auxin-mediated, auxin treatments occurred, auxin for 24, wild-type, heat-shock-inducible, histone, TF, eGFP, GFP, GAL, Gal4, CRISPR/Cas9, PROMO, JASPAR, 2B, B, P40, OT*=500, SFigure 1, CHE-1, gcy-22, CEH-36, NHR-67, OSM-3.

These pre-validation outputs illustrate several systematic sources of false positives, including metadata and author-derived strings (author names, ORCID identifiers, and editorial artifacts), cell and neuron class labels misclassified as genes (e.g., ASE, ASER, ADF, ASK, and AWC**)**, experimental conditions and generic biological descriptors (e.g., auxin, auxin-mediated, wild-type, heat-shock-inducible), and formatting artifacts and panel labels (e.g., 2B, B, P40, SFigure 1, as well as similar panel or section identifiers, construct- and tool-related strings (e.g., GFP, eGFP, GAL, Gal4, CRISPR/Cas9). These false positives arise systematically from the combination of dense captioning language, organism-specific shorthand, and the general biomedical NER training domain of these models. Validation reliably suppresses these entities, yielding a compact and interpretable gene set.

**Gene-focused false negatives introduced by validation**. While validation substantially reduces spurious gene-like strings, it can also remove biologically valid gene mentions, resulting in validation-induced false negatives. These cases arise primarily when normalization resources lack coverage for a given symbol or when conservative heuristics suppress ambiguous tokens. Concrete examples from this dataset include:

- **PMCID 3779813: nsy-5**. The gene symbol nsy-5 appears in the pre-validation gene list but is absent from the post-validation retained set, despite retention of closely related and contextually relevant genes in the same article (e.g., nsy-4, die-1, lsy-6, lin-4).
- **PMCID 4502811: epg-1**. The gene-like token epg-1 is removed during validation even though multiple canonical *C. elegans* genes with similar orthographic structure are retained.
- **PMCID 6795461: mir-58**. The miRNA-like token mir-58 is removed post-validation, whereas other miRNA identifiers (e.g., let-7, mir-84, mir-241) are retained, indicating selective loss driven by alias coverage or normalization constraints.

These examples demonstrate that validation favors precision over recall, particularly for less frequent or weakly normalized gene symbols.

**Interpretation and methodological implications.** Taken together, these results indicate that NER validation in HERMES performs two distinct but interrelated functions:

1. **Noise suppression**, by removing large volumes of non-biological or weakly supported gene-like strings and consolidating repeated mentions.
2. **Semantic triage**, by enforcing conservative acceptance criteria that prioritize high-confidence entities at the cost of reduced recall for some legitimate gene mentions.

The observed false positives and false negatives are consistent with known limitations of general biomedical NER models when applied to organism-specific, abbreviation-rich literature. Importantly, these trade-offs are explicit and intentional within HERMES: the validation step is designed to yield compact, interpretable entity sets suitable for downstream synthesis and comparison rather than exhaustive recall. Accordingly, validation rejection rates reported here should be interpreted as quantitative measures of validation impact, not as absolute error rates. Users prioritizing higher recall may inspect pre-validation outputs directly or adjust validation parameters, while default settings favor specificity and interpretability for exploratory literature mining.

**Table S5. Impact of deduplication and validation on extracted entity counts.**

| **Entity class** | **ΣN_pre (raw mentions)** | **ΣN_pre (unique)** | **ΣN_post (unique)** | **False-positive proxy rate (%)** |
| --- | --- | --- | --- | --- |
| Gene names | 6,502 | 2,304 | 751 | 67.4 |
| Cells | 4,011 | 1,014 | 82 | 91.9 |
| Organisms | 2,069 | 979 | 109 | 88.9 |
| Tissues | 686 | 278 | 71 | 74.5 |

# **Comparison of HERMES with LLM-based literature exploration tools**

To contextualize the scope and intended use of HERMES, we compared its behavior and outputs with two widely used LLM-based literature exploration tools, Elicit and Perplexity, using identical biological queries (*ASE asymmetry in Caenorhabditis elegans* and *cellulase production in Trichoderma reesei*).

**Functional scope and design philosophy.** Elicit and Perplexity are designed to support rapid, question-driven literature exploration. They typically return a limited set of highly cited or representative studies, accompanied by an LLM-generated narrative summary. These tools prioritize ease of use, conversational interfaces, and fast turnaround, but do not expose the full screened corpus, intermediate ranking scores, or raw text processing steps. Full-text access is often partial or unavailable, and results are not persisted in a reusable, machine-readable format. In contrast, HERMES is designed as a transparent, corpus-level literature mining pipeline operating exclusively on open-access PubMed Central full texts. For a given query, HERMES retrieves *all* matching PMC articles, parses and scores each article using a customizable keyword-driven relevance function, and persists the complete results table to disk. This includes per-article metadata, keyword counts, TF–IDF values, citation counts, and final scores, enabling downstream analysis with custom scripts or data science workflows. HERMES additionally generates a structured PDF report containing ranked article tables, hyperlinks to source articles, summary figures, LLM-based summaries of top-ranking papers, and validated biomedical named entities, facilitating rapid human assessment of whether a literature search has been fruitful.

**Benchmarking against Elicit.** For the “ASE asymmetry C. elegans” query (“What is known of ASE asymmetry in *C. elegans* and the role of *che-1* and *lsy-6*?”), Elicit scanned approximately **497 sources** and returned **25 selected studies**, of which **8 had full text available** in free mode. Manual expert evaluation identified the majority of returned studies as relevant, but the tool did not expose how excluded studies were ranked or filtered, nor did it allow reuse of intermediate results. In comparison, HERMES screened **371 full-text PMC articles**, saved the complete scored corpus to disk, and returned a ranked subset in which **9/10 articles in strict mode** and **9/25 in default mode** were judged to be highly pertinent. For the “cellulase production in T. reesei” query (“What is known about cellulase production in *Trichoderma reesei* and the role of *xyr1* and *cbh1*?”), Elicit scanned approximately 497 sources and returned **25 curated studies** spanning several decades of research. HERMES screened **4,459 full-text articles**, with **20/25 default-mode** and **19/25 strict-mode** top results deemed highly relevant by expert evaluation. In both cases, HERMES captured a broader and more exhaustive set of primary literature while enabling fine-grained control over inclusion criteria.

**Benchmarking against Perplexity.** Perplexity returned **10 cited sources** per query, typically emphasizing well-known reviews or highly cited primary studies. While useful for rapid orientation, this limited output did not support systematic screening or exclusion of irrelevant literature. As with Elicit, Perplexity does not provide access to the screened corpus, scoring logic, or reusable result tables. HERMES complements this approach by enabling **systematic, reproducible screening** of the entire open-access corpus matching a query, with explicit ranking and filtering rules that can be inspected, adjusted, or disabled.

**Overlap between tools.** Because Elicit and Perplexity return **limited, curated citation sets** rather than a scored corpus, comparisons were performed at the **top-K** level. Overlap was computed using PMID matching where available, and otherwise by conservative bibliographic matching (title/author/year) mapped back to PMIDs in the HERMES output lists:

ASE asymmetry in C. elegans:

- **Elicit (n = 25 included studies)** vs **HERMES default (n = 25)**: **4 shared papers** (PMIDs **34908528**, **28422646**, **21698137**, **24065887**). Overlap rate: **4/25 = 16%** (relative to HERMES) and **4/25 = 16%** (relative to Elicit).
- **Elicit (n = 25)** vs **HERMES strict mode (n = 11)**: **2 shared papers** (PMIDs **21698137**, **24065887**). Overlap rate: **2/11 = 18.2%** (relative to HERMES strict) and **2/25 = 8%** (relative to Elicit).
- **Perplexity (n = 10 cited sources)** vs **HERMES default (n = 25)**: **5 shared papers** identifiable in the HERMES top set (PMIDs **33002421**, **24065887**, **34908528**, **40586702**, **39282255**). Overlap rate: **5/10 = 50%** (relative to Perplexity) and **5/25 = 20%** (relative to HERMES default).
- **Perplexity (n = 10)** vs **HERMES strict mode (n = 11)**: **4 shared papers** (PMIDs **33002421**, **24065887**, **40586702**, **39282255**). Overlap rate: **4/11 = 36.4%** (relative to HERMES strict) and **4/10 = 40%** (relative to Perplexity).

Cellulase production in T. reesei:

- **Elicit (n = 25 included studies)** vs **HERMES default (n = 25)**: **5 shared papers** could be matched by bibliographic information and mapped to HERMES PMIDs (**36528622**, **25909478**, **26909077**, **22080343**, **29229981**). Overlap rate: **5/25 = 20%** (relative to HERMES) and **5/25 = 20%** (relative to Elicit).
- **Elicit (n = 25)** vs **HERMES strict mode (n = 25)**: **3 shared papers** could be matched and were present in the strict-mode top-25 (PMIDs **25909478**, **22080343**, **36528622**). Overlap rate: **3/25 = 12%** (relative to HERMES strict) and **3/25 = 12%** (relative to Elicit).
- **Perplexity (n = 10 cited sources)** vs **HERMES default (n = 25)**: **5 shared papers** identifiable by title/PMID (PMIDs **36376415**, **32877410**, **32765463**, **28557371**, **36528622**). Overlap rate: **5/10 = 50%** (relative to Perplexity) and **5/25 = 20%** (relative to HERMES default).
- **Perplexity (n = 10)** vs **HERMES strict mode (n = 25)**: **6 shared papers** (PMIDs **36376415**, **32877410**, **32765463**, **28557371**, **36528622**, **38187093**). Overlap rate: **6/10 = 60%** (relative to Perplexity) and **6/25 = 24%** (relative to HERMES strict).

**Speed and throughput comparison.** Runtime behavior differed substantially between HERMES and LLM-based tools due to differences in scope and computational objectives. Elicit and Perplexity prioritize **fast time-to-answer**, returning results within seconds to minutes. In our tests, Elicit required approximately **10 minutes** to process several hundred sources and return a curated list of 25 studies, while Perplexity returned approximately 10 cited sources within seconds to a few minutes. However, neither tool exposes detailed timing, throughput, or internal processing metrics. HERMES performs **explicit full-text retrieval, parsing, scoring, summarization, and reporting** across the entire retrieved corpus, resulting in longer absolute runtimes but predictable and measurable scaling. For the T. reesei query (4,459 articles), total wall-clock runtime decreased from **9,826 s (163.8 min)** with one worker to **2,700 s (45.0 min)** with 10 workers. For the C. elegans query (371 articles), runtime decreased from **3,385 s (56.4 min)** to **1,026 s (17.1 min)** under the same configuration. Increasing concurrency beyond 10 workers did not yield further gains due to external constraints, primarily NCBI Entrez rate limits and network I/O. When normalized by workload, HERMES processes **hundreds to thousands of full-text articles per run**, whereas Elicit and Perplexity return a small, fixed number of citations without exposing corpus coverage. Thus, while LLM-based tools offer faster absolute response times for exploratory queries, HERMES provides higher **per-article processing throughput** and complete transparency for systematic literature mining.

**Summary of trade-offs.** In summary, Elicit and Perplexity are well-suited for **rapid, high-level exploration** and hypothesis generation, while HERMES is optimized for **exhaustive, reproducible full-text screening**, negative filtering via strict mode, and downstream reuse of mining results. These tools are therefore best viewed as complementary rather than competing, with HERMES filling a niche not addressed by current LLM-centric literature exploration platforms.
